# Theory of temporal pattern learning in echo state networks

**DOI:** 10.1093/pnasnexus/pgag198

**Published:** 2026-05-31

**Authors:** Vincent Hakim, Alain Karma

**Affiliations:** Laboratoire de Physique de l’Ecole Normale Supérieure, CNRS, Ecole Normale Supérieure, PSL University, Sorbonne Université, Université Paris-Cité, Paris, France; Physics Department and Center for Interdisciplinary Research in Complex Systems, Northeastern University, Boston, MA, USA

**Keywords:** neuronal dynamics, learning, neuromorphic computing, artificial neural networks

## Abstract

Echo state networks are well-known for their ability to learn temporal patterns through simple feedback to a large recurrent network with random connections. However, the learning process itself remains poorly understood. We develop a quantitative theory that explains learning in a regime where the network dynamics is stable and the feedback is weak. We show that the dynamics is governed by a finite number of master modes whose nonlinear interactions can be described by a normal form. This formulation provides a simple picture of learning as a Fourier decomposition of the target pattern with amplitudes determined by nonlinear interactions that, remarkably, become independent of the network randomness in the limit of large network size. We further show that the description extends to moderate feedback and recurrent networks with multiple unstable modes.

Significance Statement Recurrent networks, key to disciplines from statistical physics to AI and neuroscience, underpin the powerful paradigm of reservoir computing. Prototypical example are the echo state network (ESN), which generate rich temporal dynamics via feedback. Despite empirical success, their theoretical basis remains poorly understood. We identify a tractable regime enabling rigorous analysis of ESN performance, bridging intuitive and mathematical understanding. Our framework extends beyond this regime, offering semiquantitative predictions and general design principles. These insights offer a significant step toward a general theory of ESNs, with implications for physics, engineering and biology, specially neuroscience.

## Introduction

Twenty years ago, Jaeger and Haas (1) presented a remarkable way of mimicking an unknown nonlinear dynamical system that outputs a given time series. They showed that feeding back into the network an appropriate linear combination of its unit activities can drive it to produce any temporal pattern. Echo state networks (ESNs) form an interesting subclass of reservoir computing (2) which has given rise to a very active engineering field (3, 4) as well as to much further research in neuroscience. In this latter context, it was shown that the appropriate feedback learning could be produced “online” by comparing at each time the output of the network with the signal (5, 6). This has led to a flourishing activity in which the training of dynamical networks helps to understand the dynamics of real biological neural networks (7). However, the principles that underlie the success of ESNs in learning a variety of temporal patterns, have remained incompletely understood, despite several previous attempts, eg (8–11). Here, we analyze the prototypical ESN of *N* recurrent units described by


(1) $$\frac{dx_{i}}{dt}=-x_{i}+g\sum_{j}M_{ij}r(x_{j})+b_{i}\;z(t),$$

where $x_{i}$ quantifies the activity of the *i*th unit, M is a Gaussian random matrix with nondiagonal elements of SD $1/\sqrt{N}$ and with zero diagonal elements corresponding to no self-interactions. The nonlinear “rate” function r(x) measures the output of each unit, taken here, as usual, to be tanh(x) for simplicity. The linear combination of outputs $z(t)=\sum_{j}w_{j}r[x_{j}(t)])$ serves both as a readout of the recurrent neural network (RNN) activity and as a feedback on the network dynamics through the fixed random vector b with elements $b_{i}$ of order one ^a^. Learning consists in computing the weights $w_{j}$ such that a desired periodic function f(t) of period *T* is reproduced, as best as possible, by the readout z(t), autonomously produced by the network dynamics of Eq. 1. One usual way (1) of computing the weights $w_{j}$ is to minimize the loss function


(2) $$S(w)=\langle [f(t)-z(t){]}^{2}\rangle_{T}+\rho \sum_{i}w_{i}^{2},$$

where the brackets denote time averaging over one period, $\langle F(t)\rangle_{T}\equiv \int_{0}^{T}F(t)\;dt/T$, and the second term on the right-hand side (r.h.s.) is a L2 regularization that controls the magnitude of the weight vector w . The weights corresponding to the unique minimum of S(w) satisfy the linear equation


(3) $$\sum_{i}C_{ij}w_{j}+\rho w_{i}=\langle f(t)r[x_{i}(t)]\rangle_{T},$$

where C is the correlation matrix between the rates of the network units, namely $C_{ij}=\langle r[x_{i}(t)]r[x_{j}(t)]\rangle_{T}$.

While different approaches have been developed to compute w (1, 6), we use here the simplest one (1). The correlation matrix C and the r.h.s. of Eq. 3 are evaluated by using, instead of the autonomous dynamics x(t), the forced dynamics $\bar{x}(t)$ obtained by replacing z(t) by f(t) in Eq. 1, implicitly assuming that learning is successful (z(t)=f(t)). Averaging is performed over one period of the forced dynamics, once the forced periodic regime is reached. Note therefore, that the initial condition of the forced dynamics does not play any role and that averaging over several periods would be redundant. We then determine w by solving the linear Eq. 3 and run the autonomous dynamics of Eq. 1 with $z(t)=\sum_{j}w_{j}r[x_{j}(t)])$ as a self-consistency check of this assumption. This assumption turns out to be satisfied for all present computations that use a small regularization ($\rho =10^{-10}$) ensuring that the cost function is minimized for z(t) very close to f(t). Importantly for what follows, even when z(t)≈f(t) over one period, the learned nonlinear limit cycle of the autonomous dynamics can itself be linearly stable or unstable with z(t) remaining close to or deviating from f(t), respectively, over several periods.

A classic result of random matrix theory states that the eigenvalues of large matrices M are uniformly distributed in the unit disk (13) so that the dynamics without feedback is stable for g<1 while it has many unstable modes for g>1 and is actually chaotic (14, 15). To understand the learning process, we focus first on a regime where the dynamics without feedback is linearly stable (g<1) and the feedback is weak. The feedback in Eq. 1 is the product of z(t)=f(t) and $b_{i}$ and only the amplitude of this product matters. We choose here to keep the components of b of order one and take f(t)≪1. Equivalently, if one prefers to consider functions of order one, one could take $b_{i}\ll 1$. This small feedback regime is made analytically tractable by the fact that the learned dynamics is slaved to a few small amplitude “master modes,” which weakly interact nonlinearly to produce stable or unstable limit cycles. As a result, the dynamics of Eq. 1 can be reduced to the evolution equations for the complex amplitudes of those modes using a normal form approach dating back to Poincaré and widely used to reduce the dimensionality of nonlinear dynamical systems (16–20), here from N≫1 to a small number of modes.

## Results

**Figure 1:**
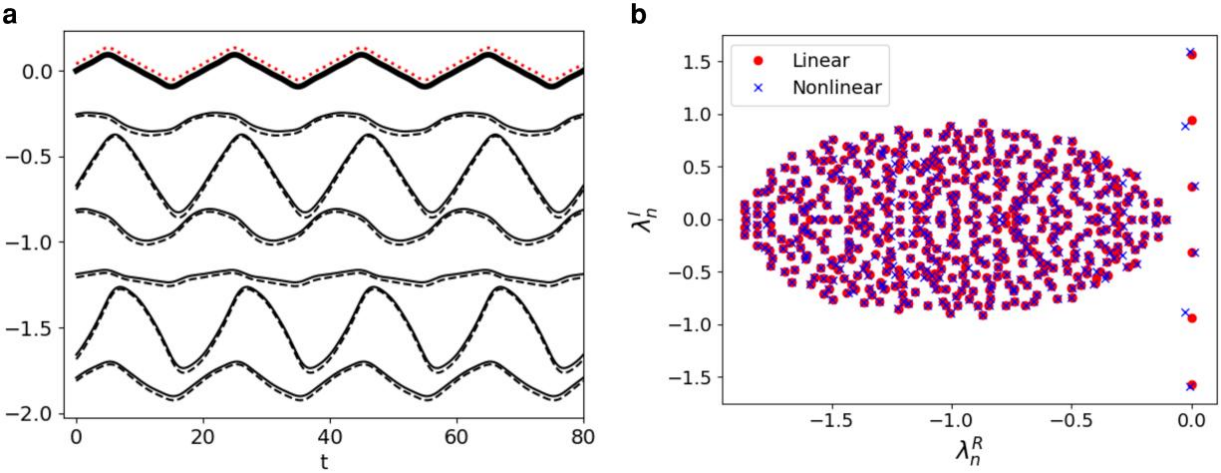
An example of ESN learning in the stable (g=0.9) network regime. a) The autonomous dynamics of the network (thick solid dark line) reproduces after learning the target “sawtooth” (red dotted line slightly shifted upward for visibility). Examples of network unit activities are also shown both for the nonlinear (thin solid dark lines) and linear (dashed dark lines slightly shifted upward for visibility) cases. b) Spectrum of the associated linear dynamics (N=500) both in the nonlinear (red circles) and linear (blue crosses) cases. Note the few separated eigenvalues with zero (linear case) or small (nonlinear case) real parts, which correspond to the frequencies of the sawtooth. Here and in the following figures, Eq. 3 for both forced and autonomous dynamics is solved using a fourth-order Runge–Kutta algorithm with dt=0.1, and, for simplicity, the feedback vector b is taken to have identical components on all units, b=(1,…,1).

To illustrate the existence of these master modes, we first compare in Fig. 1 the results of learning a truncated Fourier sine series of a sawtooth function f(t)=A[sin(ωt)−sin(3ωt)/9+sin(5ωt)/25] (A=0.1 and ω=π/10) with both the nonlinear dynamics of interest, r(x)=tanh(x), and its linear approximation, r(x)=x; these two choices produce different w due to the presence and absence of nonlinearity through r(x) in Eqs. 1 and 3, respectively. However, they produce almost indistinguishable autonomous network dynamics (Eq. 1 with $z(t)=\sum_{j}w_{j}r[x_{j}(t)])$) reproducing the target sawtooth (Fig. 1a). They also produce very similar stability spectra of the autonomous dynamics of Eq. 1 linearized around x=0, corresponding to dx/dt=Lx where


(4) $$L\equiv -I_{N}+gM+bw^{T},$$

and $I_{N}$ denotes the N-dimensional identity matrix. As shown in Fig. 1b, the spectrum of the matrix L with w learned with r(x)=x displays three pairs of complex conjugate eigenvalues with vanishing real parts and imaginary parts exactly equal to the frequencies $\omega_{n}=\pm n\omega ,\;(n=1,3,5)$ of the sawtooth. In this case, the autonomous dynamics is purely linear and any superposition of these six modes is a solution of the autonomous dynamics consistent with the findings of previous studies (21–23). The sawtooth function is obtained by precisely choosing the amplitudes of these six modes to produce the desired output superposition, $z(t)=\sum_{n}Z_{n}\exp\;(i\omega_{n}t)$, where the $Z_{n}$ match the amplitudes $f_{n}$ of the target function $f(t)=\sum_{n}f_{n}\exp\;(i\omega_{n}t)$, as detailed in the Materials and methods section. As a result of linearity (r(x)=x), the autonomous dynamics is marginally stable, ie the linear operator L has eigenvalues with real parts equal to zero, since otherwise the amplitude of x would either blow up or vanish, and the function f(t) could not be reproduced. As a result, any perturbation changes the amplitudes $Z_{n}$ and thus changes the output function z(t). In contrast, with nonlinearity (r(x)=tanh(x)), one or more pairs of complex conjugate eigenvalues have a small positive real part and $Z_{n}\approx f_{n}$ must emerge as a fixed point of the slow dynamics that governs the evolution of the complex amplitudes $Z_{n}(t)$ over a time scale larger than *T*. Understanding learning is now reduced to answering the basic questions: What is the relation between the linear dynamics and the nonlinear dynamics ? How do fixed points emerge? What controls their stability? Answering these questions is the main aim of the present work.

**Figure 2:**
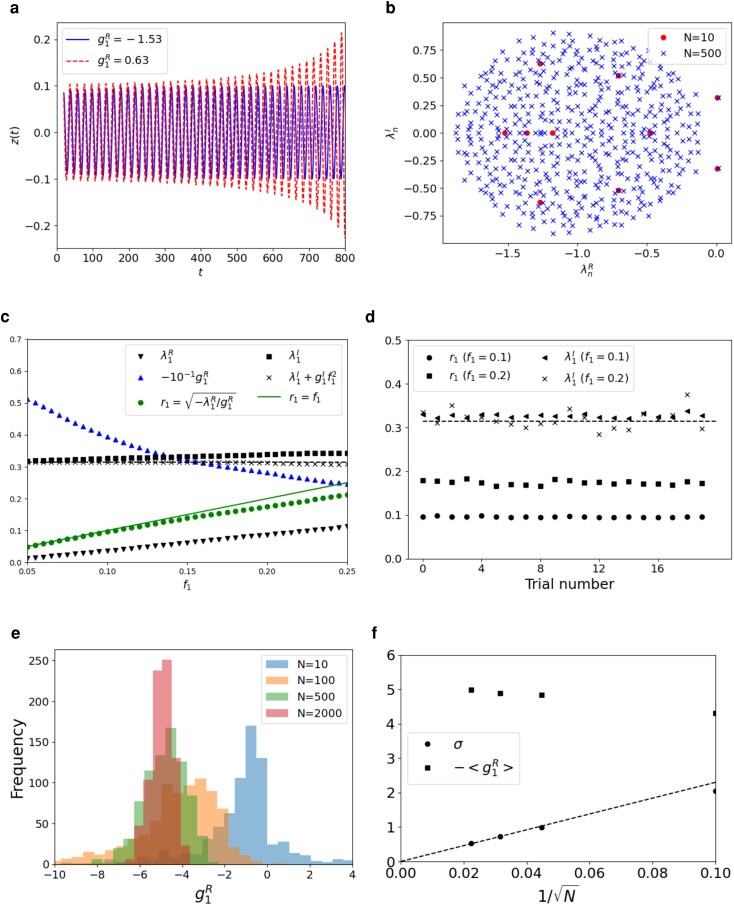
Learning a single cosine function $2f_{1}\cos\;(\omega t)$ (ω=π/10) in the stable network regime (g=0.9). a) Two examples of autonomous dynamics after learning ($f_{1}=0.05$, N=10) which can be stable (blue solid line) or unstable (red dashed line). The real part of $g_{1}^{R}$ (Eq. 7) is indicated in each case. b) Examples of L-spectra after learning for a small (N=10) and a large network (N=500). c) For a given matrix M, it is displayed after learning, as a function of the half-amplitude $f_{1}$, the real and imaginary parts of the eigenvalue $\lambda_{1}$ and the coefficient $g_{1}$ (Eq. 7, as well as the predicted amplitude of the autonomous limit cycle $r_{1}$ and its nonlinearly corrected frequency (Eq. 8). The amplitude of the read-out z(t) after learning is indistinguishable from $f_{1}$ (solid green line). d) Predicted amplitude $r_{1}$ and noncorrected frequency $\lambda_{1}^{I}$ of the autonomous dynamics after learning for a set of 20 matrices M, for two amplitudes $f_{1}$. e) Histogram of the values of the real part $g_{1}^{R}$ for different network sizes. f) Mean value $\left\langle g_{1}^{R}\right\rangle$ and SD *σ* of the histogram in e) as a function of network sizes.

The simplest possible setting to address these questions is provided by the case of a single frequency, $f(t)=f_{1}\exp\;(i\omega t)+c.c$., where c.c. denotes complex conjugation. It is illustrated in Fig. 2a for a pure cosine ($f_{1}=f_{1}^{*}$) of amplitude $2f_{1}$  ^b^. The linear operator L after learning with the nonlinear rate function (Fig. 2b), has two slow modes with complex conjugate eigenvalues equal to $\lambda_{1}$ and $\lambda_{1}^{*}$, close to but different from ±iω. We show below that this difference is governed by the amplitude of the feedback and is of the order of $\sqrt{\left|f_{1}\right|}$. Together with the nonlinear interaction between the slow modes, it determines the characteristics of the limit cycle. In order to see it, the network activity at the linear level is taken to be $x(t)=A_{1}(t)\exp\;(i\omega t)\;u_{1}+c.c$. where $A_{1}$ is arbitrary and $u_{1}$ is the right eigenvector of L corresponding to the eigenvalue $\lambda_{1}$. At the next nonlinear order, the slow evolution of $A_{1}(t)$ is determined by the classical Poincaré–Hopf normal form. It is obtained from the nonappearance of resonant terms in the dynamics (16, 17, 19, 20), which yields the amplitude equation


(5) $$\frac{dA_{1}}{dt}=(\lambda_{1}-i\omega )A_{1}+g_{A}|A_{1}{|}^{2}A_{1},$$

where $A_{1}$ evolves slowly on the time scale of the period *T* since $|\lambda_{1}-i\omega |\ll 1$ when $|f_{1}|\ll 1$ (*ω* itself is not supposed to be small). The form of Eq. 5 is determined by the symmetries of the full network dynamics (Eq. 1). Namely, Eq. 5 is the lowest order equation invariant under time translation, $A_{1}\to \exp\;(i\phi )A_{1}$ and under the reflection x→−x, which translates into $A_{1}\to -A_{1}$. The value of the constant $g_{A}$ is obtained as,


(6) $$g_{A}=3r_{3}(\lambda_{1}+1)\sum_{\;j}v_{1,j}|u_{1,j}{|}^{2}u_{1,j},$$

where $u_{1}$ and $v_{1}$ are right and left eigenvectors of L for the eigenvalue $\lambda_{1}$, with the normalization $v_{1}^{T}u_{1}=1$, and $r_{3}=-1/3$ is the coefficient of the cubic nonlinearity of $r(x)=\tanh\;x=x+r_{3}x^{3}+\cdots$ as detailed in Materials and methods. The evolution of $z(t)=Z_{1}(t)\exp\;(i\omega t)+c.c$, is obtained in turn by projecting the network activity with the readout vector w,


(7) $$\frac{dZ_{1}}{dt}=(\lambda_{1}-i\omega )Z_{1}+g_{1}|Z_{1}{|}^{2}Z_{1},\;g_{1}=g_{A}/|w^{T}u_{1}{|}^{2}$$

The real and imaginary part of the linear dynamics eigenvalue, $\lambda_{1}^{R}$ and $\lambda_{1}^{I}$ and of the Poincaré–Hopf coefficient $g_{1}$, ie $g_{1}^{R}$ and $g_{1}^{I}$ determine whether Eq. 7 possesses a limit cycle and what its amplitude $r_{1}$ and frequency $\delta \omega_{1}$ are. Namely when $\lambda_{1}^{R}g_{1}^{R}<0$, these are


(8) $$r_{1}=\sqrt{-\lambda_{1}^{R}/g_{1}^{R}},\;\delta \omega_{1}=\lambda_{1}^{I}-\omega +g_{1}^{I}r_{1}^{2}.$$

For learning the correct fixed point, independent of its stability discussed below, the choice of w which controls both $\lambda_{1}$ and $g_{1}$, should be such that the output z(t) exactly matches the target pure cosine ($f_{1}=f_{1}^{*}$) of amplitude $2f_{1}$ and frequency *ω*, which implies the two equalities $r_{1}=f_{1}$ and $\delta \omega_{1}=0$.

To obtain the coefficient $g_{1}$ and test these results, we computed the left and right eigenvectors, $u_{1}$ and $v_{1}$, of the matrix L with w solution of Eq. 3. The results are illustrated in Fig. 2c for a given matrix M and cosine functions of different amplitudes $2f_{1}$. The slow mode eigenvalue $\lambda_{1}$ starts from ±iω for a vanishing $f_{1}$ and has a growing real part as $f_{1}$ increases. The corresponding coefficient $g_{1}$ also varies with $f_{1}$ and combines with $\lambda_{1}^{R}$ such that the resulting amplitude $r_{1}$ of the autonomous limit cycle matches $f_{1}$, in accordance with Eq. 8. This is also true for the frequency of the limit cycle. As shown in Fig. 2c, the nonlinear departure of the frequency from *ω* due to the imaginary part $g_{1}^{I}$ of $g_{1}$ is compensated by the departure of the imaginary part of $\lambda_{1}^{I}$ from *ω* so that $\delta \omega_{1}≃0$. The close agreement between the predicted amplitude $r_{1}$ of the limit cycle and the desired amplitude $f_{1}$ is observed independently of the chosen matrix M as illustrated in Fig. 2d.

The above conditions on $r_{1}$ and $\delta \omega_{1}$ ensure that the proper feedback has been learned, such that the autonomous dynamics displays a limit cycle of the right amplitude and frequency, meaning that when the initial activity exactly stands on the limit cycle, the desired sinusoidal function will be produced. However, this does not ensure that the limit cycle is a stable attractor of the autonomous dynamics, namely that the network activity returns to the desired periodic behavior when the limit cycle is perturbed or when starting with initial condition slightly away from it (Fig. 2a). In this one-frequency case, the existence of a limit cycle (Eq. 8) requires the real parts $\lambda_{R}$ of the slow mode and of $g_{1}$, $g_{1}^{R}$, to be of opposite signs. Stability of the limit cycle simply corresponds to $\lambda_{R}>0,g_{1}^{R}<0$ whereas for $\lambda_{R}<0,g_{1}^{R}>0$ any small perturbation of the amplitude $A_{1}$ makes it grow away from $f_{1}$. Learning can in principle produce both cases. As illustrated in Fig. 2a for networks of size N=10, starting from randomly drawn matrices M, autonomous dynamics with unstable limit cycles are often produced. However, for networks sizes of 100 or higher, quite remarkably, instability is almost never observed. In order to better understand this surprising growing success of limit cycle stability with the network size, we computed the distribution of Poincaré–Hopf coefficients $g_{1}$ given by Eq. 7 for ensemble of random matrices of different sizes *N*. The corresponding histograms of the real part $g_{1}^{R}$ of $g_{1}$ are shown in Fig. 2e. For N=10, a significant fraction of matrices M produces positive $g_{1}^{R}$, corresponding to unstable limit cycles. However, as *N* becomes larger, the histogram means become more negative and the histograms shrink around their negative mean value. As quantified in Fig. 2f, the SD of $g_{1}$ decreases as $1/\sqrt{N}$ around a mean value that extrapolates for N→+∞ to the negative value $g_{1}^{\left(\infty \right)}≃-5.2$, for $f_{1}=0.05$. This concentration of the histograms around their means, together with the shift of the histogram means toward more negative values, explains why positive Poincaré–Hopf coefficients are less and less frequently found as *N* grows. Although, the results of Fig. 2e and f are shown for $f_{1}=0.05$, we generally observed this behavior for all amplitudes in the range $0\leq f_{1}\leq 0.25$. When $f_{1}\to 0$, the asymptotic coefficient $g_{1}^{\left(\infty \right)}$ for N→∞, can actually be directly computed from the explicit solution of learning with a linear activation, as detailed in the Materials and methods section. This gives


(9) $$g_{1}^{\left(\infty \right)}=6r_{3}\frac{1+i\omega }{1+\omega^{2}-g^{2}}\;\frac{b^{T}b}{N}.$$

It is worth noting that the remarkable stability of the learned dynamics for large networks which holds independently of the considered random network, depends on the saturating character of the tanh cubic nonlinearity, $r_{3}<0$, as shown by Eqs. 6 and 9. For a rate activation with $r_{3}>0$ on the contrary almost no limit cycle would be stable for large network sizes.

**Figure 3:**
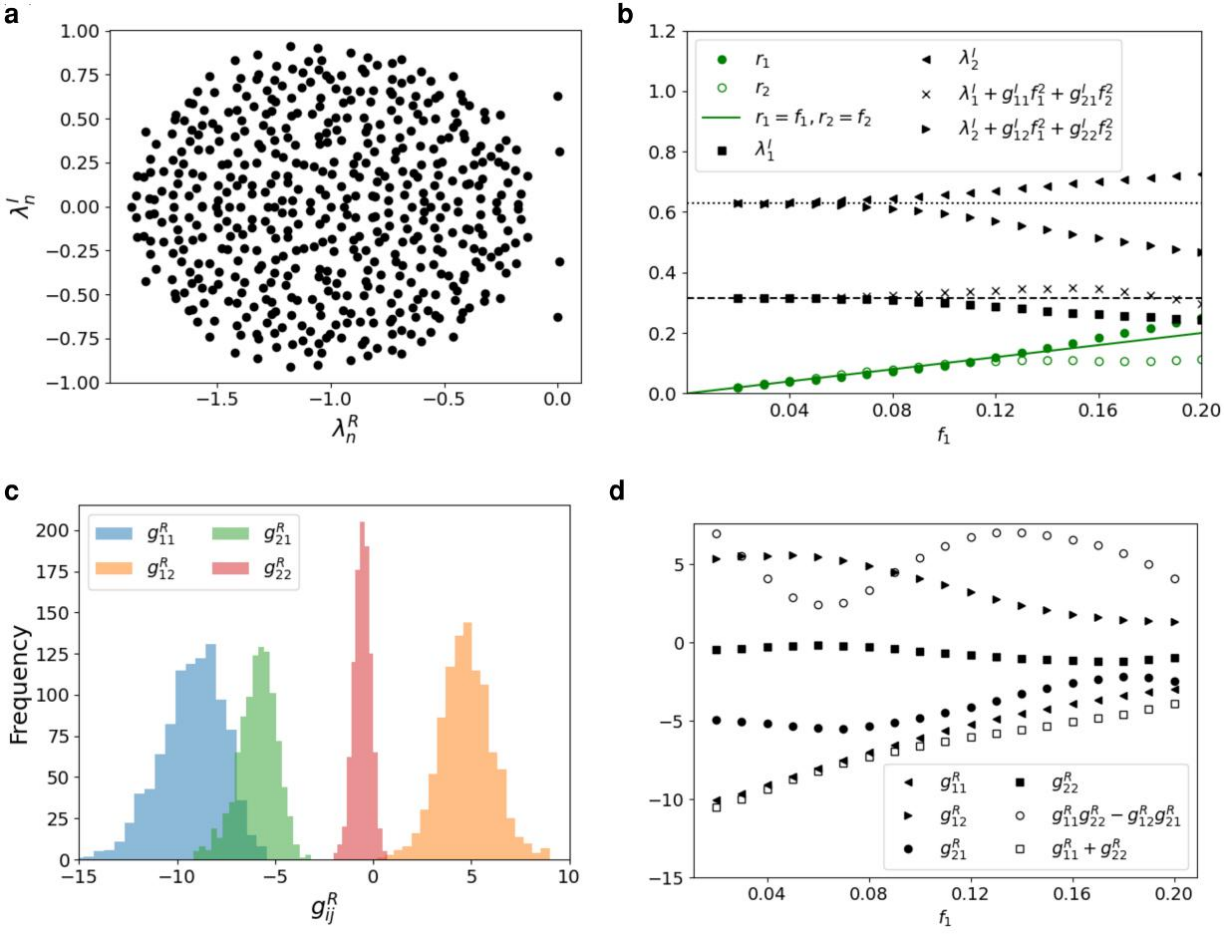
Learning a two-frequency function $f(t)=2f_{1}[\cos\;(\omega t)+\cos\;(2\omega t)]$ with ω=π/10. a) Example of L -spectrum (Eq. 4) after learning ($f_{1}=0.025,N=500$). b) Results of learning for different amplitudes $f_{1}$ for the same matrix M as in a). The amplitudes $r_{1}$ and $r_{2}$ as obtained from Eq. 12 are shown as well as the imaginary parts $\lambda_{1}^{I}$ and $\lambda_{2}^{I}$ of the two dominant eigenvalues, together with the corresponding nonlinearly corrected imaginary parts (Eq. 13). The target frequencies *ω* (dashed line) and 2ω (dotted line) are also shown. c) Histograms of the real parts of the coefficients $g_{ij}$ (Eq. 28) obtained for learning with $f_{1}=0.025$, N=500, and 200 different matrices M. d) The real parts of the coefficients $g_{ij}$ are shown for different amplitudes $f_{1}$, together with the limit-cycle stability conditions (Eq. 14) (same simulations as in b)). Note that the stability conditions (Eq. 14) are always satisfied.

The theory is easily applied to describe the learning of more complex functions with a finite number of frequencies, as the sawtooth of Fig. 1. We treat here first the case of a general two-frequency function $f(t)=[f_{1}\exp\;(i\omega t)+f_{2}\exp\;(i2\omega t)+c.c]$ with the case $f_{1}=f_{1}^{*}=f_{2}=f_{2}^{*}$ illustrated in Fig. 3. In this case, learning with a linear rate function with a vanishingly small regularization produces an operator L with four slow modes with imaginary parts equal to ±iω and ±i2ω. Learning from the full Eq. 3, displaces the two pairs of eigenvalues to $\lambda_{1},\lambda_{1}^{*}$ and $\lambda_{2},\lambda_{2}^{*}$, as shown in Fig. 3a. The network output function is described as $z(t)=[Z_{1}\exp\;(i\omega t)+Z_{2}\exp\;(i2\omega t)+c.c]$ with $Z_{1}$ and $Z_{2}$ “slow” time-dependent functions, the dynamics of which depends both on the displaced linear eigenvalues and the nonlinear interactions between the slow modes. Their evolution is governed by the following normal form,


(10) $$\frac{dZ_{1}}{dt}=(\lambda_{1}-i\omega )Z_{1}+(g_{11}|Z_{1}{|}^{2}+g_{21}|Z_{2}{|}^{2})Z_{1}$$

(11) $$\frac{dZ_{2}}{dt}=(\lambda_{2}-i2\omega )Z_{2}+(g_{12}|Z_{1}{|}^{2}+g_{22}|Z_{2}{|}^{2})Z_{2}$$

with the constants $g_{ij}$ given by expressions similar to Eq. 7 for $g_{1}$, as detailed in Materials and methods. As in the previous example (Eq. 7), the cubic lowest nonlinear terms that appear are restricted by symmetries, namely time translation invariance, $Z_{1},\;Z_{2}\to \exp\;(i\phi )Z_{1},\;\exp\;(i2\phi )Z_{2}$, and the inversion, $Z_{1},Z_{2}\to -Z_{1},-Z_{2}$ coming from the use of tanh activation in Eq. 1.

A limit cycle with amplitudes $r_{1}=|Z_{1}|$ and $r_{2}=|Z_{2}|$ exists when the following conditions are satisfied,


(12) $$\lambda_{1}^{R}=-g_{11}^{R}r_{1}^{2}-g_{21}^{R}r_{2}^{2},\;\lambda_{2}^{R}=-g_{12}^{R}r_{1}^{2}-g_{22}^{R}r_{2}^{2}.$$

In order to reproduce the target function f(t), the solutions $r_{1}$ and $r_{2}$ of Eq. 12 should obey $r_{1}=|f_{1}|$, $r_{2}=|f_{2}|$. In order to obtain the correct frequencies of the two modes, the imaginary parts of linear modes $\lambda_{1}^{I}$ and $\lambda_{2}^{I}$ should moreover compensate the nonlinear frequency corrections,


(13) $$\lambda_{1}^{I}+g_{11}^{I}|f_{1}{|}^{2}+g_{21}^{I}|f_{2}{|}^{2}=\omega ,\;\lambda_{2}^{I}+g_{12}^{I}|f_{1}{|}^{2}+g_{22}^{I}|f_{2}{|}^{2}=2\omega .$$

For large *N* (ie N=500), learning is almost always successful and both the amplitude and phase conditions are found to be well-obeyed, as illustrated in Fig. 3b for a given matrix M and varying amplitude. As in the simple case of the cosine function (Eq. 7), Eqs. 12 and 13 are not sufficient to ensure the stability of the autonomous dynamics limit cycle. Taking $Z_{1}=r_{1}\exp\;(i\phi_{1})$ and $Z_{2}=r_{2}\exp\;(i\phi_{2})$, a simple analysis shows that the fixed point amplitudes $r_{1}=f_{1}$ and $r_{2}=f_{2}$ are linearly stable when the two following conditions are obeyed


(14) $$g_{11}^{R}r_{1}^{2}+g_{22}^{R}r_{2}^{2}<0,\;g_{11}^{R}g_{22}^{R}-g_{12}^{R}g_{21}^{R}>0.$$

Quite remarkably for N=500 or larger, the limit cycle is found to be always stable ^c^ and these relations always satisfied as shown in Fig. 3c for an ensemble of matrices M for the same two-cosine function f(t), and in Fig. 3d for the same matrix M and varying the amplitude of this function. As in the simpler single frequency case, this results from the $g_{ij}$ coefficients tending to well-defined limiting values in the large network size limit, independently of the random network interaction matrix M. In particular, the limiting values of $g_{11}^{R}$ and $g_{22}^{R}$ are negative while $g_{12}^{R}>0$ and $g_{21}^{R}<0$ are of opposite signs, ensuring the two conditions 14 for any values of the limit cycle amplitudes $f_{1}$ and $f_{2}$. Of note, these limiting values ensure that $\lambda_{1}^{R}$ is positive and can drive the growth of the limit cycle.

**Figure 4:**
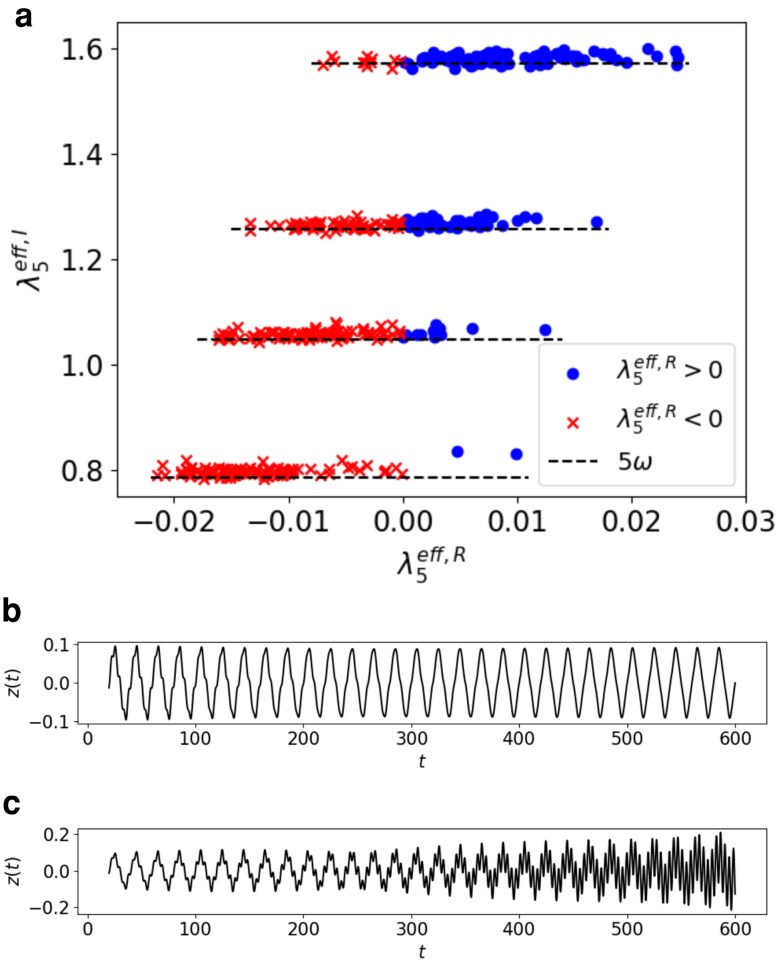
Prediction of the stability of learned nonlinear attractors for the three-frequency sawtooth. a) Plot of $\lambda_{5}^{\mathrm{eff}}$ (imaginary part $\lambda_{5}^{\mathrm{eff},I}$ versus real part $\lambda_{5}^{\mathrm{eff},R}$) for the sawtooth function of Fig. 1 defined by f(t)=A[sin(ωt)−sin(3ωt)/9+sin(5ωt)/25] with A=0.1, g=0.9, and N=500. The results are shown for four different frequencies corresponding to ω=2π/T for T=20, 25, 30, and 40, and 100 different matrices M for each period, corresponding to each symbol. The values of $\lambda_{5}^{\mathrm{eff},I}$ line up as expected with the dashed lines corresponding to $\lambda_{5}^{\mathrm{eff},I}=5\omega$ for the four different *T* values. Equation 15 predicts that the learned sawtooth is linearly stable for $\lambda_{5}^{\mathrm{eff},R}<0$ (red crosses) or unstable for $\lambda_{5}^{\mathrm{eff},R}>0$ (blue circles). This prediction is illustrated for T=20 for one of the stable case b), where a small perturbation of the attractor decays over several periods, and one of the unstable case c) where the perturbation grows by amplification of the 5ω mode. The sign of $\lambda_{5}^{\mathrm{eff},R}$ correctly predicted stability for all 400 cases tested (100 matrices M for four different frequencies) with only one exception for T=40 where the sawtooth was unstable even though $\lambda_{5}^{\mathrm{eff},R}<0$. Closer examination of this case revealed that the 3ω mode, which was stable in all other cases, was unstable in this rare case (ie $\lambda_{3}^{\mathrm{eff}}\equiv Re[\lambda_{3}+g_{A,13}|A_{1}{|}^{2}]>0$). Stability is seen to depend sensitively on frequency with 12 stable cases for T=20 and 97 stable cases for T=40 (excluding the red cross with the unstable 3ω mode).

The extension of the amplitude equation framework to more than two frequencies is in principle straightforward, but the number of Landau coefficients increases rapidly with the number of frequencies. Fortunately, not all Landau coefficients need to be calculated to understand the stability of the trained nonlinear attractors in nontrivial cases. For example, for the sawtooth example of Fig. 1, there are 17 Landau coefficients that control the evolution of the three amplitudes $A_{1}$, $A_{3}$, and $A_{5}$ of the *ω*, 3ω, and 5ω modes, respectively. However, owing to the fact that the amplitudes have different magnitudes ($A_{1}\gg A_{3}\gg A_{5}$), it is possible to explain dramatic changes in the stability of learned attractors as a function of the sawtooth amplitude or frequency by computing only one Landau coefficient. This stems from the property that the stability of the entire sawtooth attractor turns out to be governed by the stability of the highest frequency 5ω mode, which is itself governed by an equation of the form


(15) $$\partial_{t}A_{5}=(\lambda_{5}-i5\omega +g_{A,15}|A_{1}{|}^{2})A_{5}+N(A_{1},A_{3}),$$

where the nonlinear term $N(A_{1},A_{3})$ depends predominantly on the amplitudes of the lower frequency modes, since $A_{5}$ is much smaller than both $A_{1}$ and $A_{3}$. Since the nonlinear term is independent of $A_{5}$, Eq. 15 implies that the stability of the sawtooth is controlled by the sign of the real part of λ5eff≡Re[λ5+gA,15|A1|2]. We have computed $g_{A,15}$ as given by the formula of Eq. 28. The resulting plot of $\lambda_{5}^{\mathrm{eff}}$ as a function of frequency *ω* is shown in Fig. 4. The change of sign of $\lambda_{5}^{\mathrm{eff}}$ predicts that a larger fraction of sawtooth attractors becomes stable with decreasing frequency in excellent quantitative agreement with numerical observations. This change of sign also correctly predicts that the fraction of stable sawtooth attractors increases rapidly with amplitude. The amplitude equation framework remains predictive for more than three frequencies. For example, we have checked that the stability of the 4-frequency sawtooth f(t)=A[sin(ωt)−sin(3ωt)/9+sin(5ωt)/25−sin(7ωt)/49] is well predicted by the sign of both $\lambda_{5}^{\mathrm{eff}}$ and $\lambda_{7}^{\mathrm{eff}}\equiv Re[\lambda_{7}+g_{A,17}|A_{1}{|}^{2}]$, which must both be negative for the sawtooth to be stable. Beyond four or five frequencies, the theory of learning within the amplitude equation framework becomes limited by the fact that the magnitude of the network output weights grows rapidly with the number of frequencies as shown in the Materials and methods (cf. Eq. 34), for the limit N→∞ and $f_{1}\to 0$. In this limit, the square norm of w is given, for M/2 complex conjugate pairs of purely imaginary eigenvalues $\pm i\sigma_{m},m=1,\ldots ,M/2$, by


(16) $$w^{T}w=\frac{g^{2}}{b^{T}b}\left[-1+\prod_{m=1}^{M/2}\frac{{\left(1+\omega_{m}^{2}\right)}^{2}}{g^{2}}\right].$$

This grows faster than exponentially with *M* for equally spaced complex pairs of eigenvalues $\omega_{m}=\pm m\omega ,m=1,\ldots ,M/2$. The maximum number of modes that can be described by amplitude equations in a nonlinear regime will generally depend on the finite amplitudes of the modes, *N*, and *ω*. The 3- and for 4-frequency sawtooth examples indicate that the above constraint on the weights is not so stringent. Another limitation of the amplitude equation framework worth noting is that, when different frequency modes have very different amplitudes as in the sawtooth examples, nonlinear terms contain higher-order contributions from linearly stable slave modes that may not always be negligible. Therefore, including the contributions of these modes can further improve the quantitative accuracy of the amplitude equations as will be discussed elsewhere.

**Figure 5:**
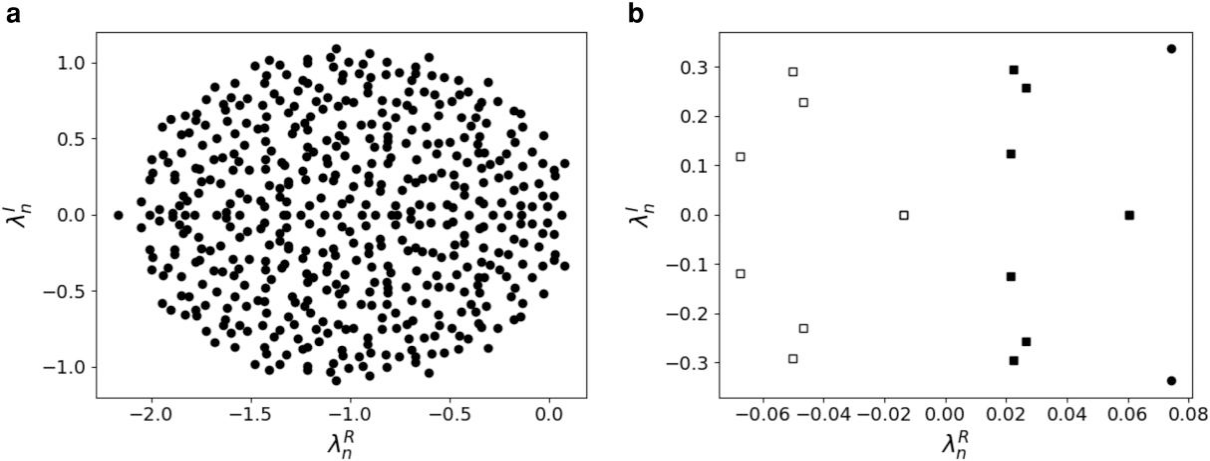
Learning in the presence of several unstable modes. a) Spectrum of L for a network with g=1.1 after learning $f(t)=2f_{1}\cos\;\omega t$ ($f_{1}=0.05,\omega =\pi /10,N=500$). b) Magnification of the unstable part of the spectrum. Nine modes have a positive real part (solid squares). Seven eigenvalues displaced by the interaction with the most unstable complex conjugate pair corresponding to $\lambda_{1}^{I}\approx \omega$ are also shown (empty squares) and all have negative real parts.

We have examined so far cases when the recurrent dynamics without feedback is stable (g<1). However, the normal form description can also be applied in the case when g>1 and the dynamics without feedback has a number of unstable modes. Figure 5a shows the whole spectrum of the matrix L when successfully learning a simple cosine function with g=1.1. It comprises a pair of unstable modes with imaginary parts close to $\lambda_{1}^{I}=\pm i\omega$ and a positive real part $\lambda_{1}^{R}≃0.08$ similarly to spectra obtained for g<1 (Fig. 2). However, for g=1.1, there are seven other linearly unstable modes as seen in the spectrum magnification (Fig. 5b). Contrary to naive expectations these modes do not prevent the stable learning of the sine function. At the weakly nonlinear level, the normal form approach developed here provides the (lowest-order) interaction between the amplitude $A_{n}$ of any of these linearly unstable modes and the amplitude $A_{1}$ of the master mode corresponding to the eigenvalue $\lambda_{1}$, yielding


(17) $$\frac{dA_{n}}{dt}=\lambda_{n}A_{n}+g_{A,1n}|A_{1}{|}^{2}A_{n},\;g_{A,1n}=6r_{3}(\lambda_{n}+1)v_{n}^{T}\left[|u_{1}{|}^{2}u_{n}\right].$$

This interaction with the master mode keeps in check the seven modes n=2,…,8, by renormalizing their eigenvalues $\lambda_{n}$ to $\lambda_{n}+g_{A,1n}|f_{1}{|}^{2}$ which have negative real parts, as shown in Fig. 5b. The eigenvalue displacements depend on the function amplitude $f_{1}$, which should be large enough to render negative the real parts of the renormalized eigenvalues for successful learning. Indeed, we found that, for g=1.1, learning fails for the matrix shown in Fig. 5 when $f_{1}\leq 0.05$ as predicted by Eq. 17. This also agrees with previous results (8, 10) showing that forcing should be strong enough to suppress chaos, as intuitively expected.

## Conclusions and outlook

In conclusion, we have shown in the present work that considering networks with stable dynamics and a small feedback/readout allows for the rational analysis of ESN. As seen in the displayed examples, the description developed for functions of small amplitudes remains semiquantitatively accurate for moderate amplitudes (eg $f_{1}=0.2$ in Figs. 2 and 3). We have moreover observed that it also remains applicable when the network interaction matrix M has several unstable modes (ie g>1, Fig. 5). It remains an interesting topic for future work to see how far the approach can be pushed into the chaotic regime. We have emphasized the case of large random matrices of relevance to biological neural networks, but our main results and methods can be applied to specific matrices of smaller sizes that are considered in some engineering applications (3). The understanding of ESN that we have developed here, may thus help the design of actual ESN. We have restricted our analysis to prototypical RNNs with Gaussian interactions and odd activation function. This has eased the analysis in two ways, first by fixing the stable network fixed point at x=0 and, second, by introducing the inversion symmetry (x→−x) that simplifies normal form computations. Although, these features are mathematically convenient, our approach should be applicable to perturbations around a nontrivial fixed point with more general activation functions. As such, it appears interesting to study various generalizations of relevance to neural dynamics eg for excitatory–inhibitory networks with more complex dynamical units and with neural connections obeying Dale’s law (12) or more structured networks with different unit classes. In the context of machine learning and dynamical system reconstruction, the prototypical ESN that we have studied has, of course, been superseded by more recent algorithms. However, learning the dynamics of a RNN remains at the core of state-of-the art algorithms (24). Besides its intrinsic interest, the analysis that we have developed here, may prove useful for the development and interpretability of these more complex algorithms and, for instance, could help in going beyond the use of piecewise linear or almost-linear networks.

The present theory should also be useful to analyze important questions that have started to be addressed experimentally (25), namely, how trajectory recovery from perturbation is eased by the nonlinear character of the movement production dynamics, as well as the required precision of the initial state produced during the preparatory period for the correct production of a precise movement. In this respect, we note that, in the amplitude equation framework, the approach to the dynamical attractor is controlled by the real part of the eigenvalues of the master mode dynamics. While this approach takes place over several periods of oscillation in the small feedback limit, it can occur on a time scale comparable or smaller than one period for moderate feedback (eg $f_{1}∼0.2$ for a single frequency), which could be physiologically relevant.

Our approach also provides a theoretical framework to understand other learning approaches beyond ESN, including those that modify network connection strengths (M) in addition to output weights (w) (6, 7, 26). The sawtooth example of Fig. 4 already illustrates how the amplitude equation approach can efficiently predict connection strengths that yield stable attractors without the need to simulate the dynamics beyond learning over one period. We further note that networks with low-rank connectivity have recently been proposed to account for neural dynamics in several contexts such as discrimination (27) or go-response (28) tasks. In effect, our analysis is complementary to these recent works in showing that feedback, in the regime we have considered, reduces the dynamics of a large random network to that of few interacting modes, namely to the dynamics of a low-rank network. It also shows how to design low-rank networks with more general dynamics than those considered so far. It will be interesting to see whether real neural systems, such as the reciprocally interacting thalamus and cortex (21), have taken advantage of this design.

## Materials and methods

### Choice of network dynamics

We have described RNN dynamics using Eq. 1, as done in Ref. (6, 7) and as usual in the computational neuroscience community. Another classical formulation focuses on the rates $y_{i}$ of the elementary units and writes the dynamics as,


(18) $$\frac{dy_{i}}{dt}=-y_{i}+r\left(\sum_{j}J_{ij}y_{j}\right).$$

The matrix J describes the interaction between the network units and in our case is composed of random interactions and a learned rank 1 component, $J=gM+bw^{T}$. It is worth noting that Eq. 1 follows from Eq. 18. In order to see it, let us assume that Eq. 18 holds and define x as Jy. Then, Eq. 18 implies that the evolution of $x_{i}$ reads


(19) $$\frac{dx_{i}}{dt}=\sum_{j}J_{ij}\frac{dy_{j}}{dt}=-x_{i}+\sum_{j}J_{ij}r(x_{j}),$$

which is seen to be identical to the autonomous dynamics of Eq. 1 after substituting $J=gM+bw^{T}$ on the right hand side of the second equality.

### Analytic description of learning in linear networks

When the rate function is linear, r(x)=x, learning can be precisely understood, as we briefly show here, building upon previous works (21–23). The problem is actually a particular case, for a linear feedback of rank 1, of the classical “pole placement” problem thoroughly studied in control theory (29, 30). We consider learning of a real function with *M* modes, counting a positive frequency mode and the associated negative one as two distinct modes (eg M=2 for the sine function, M=4 for the two frequency-function of Fig. 3 and M=6 for the truncated sawtooth function of Figs. 1 and 4). The forced solution $\bar{x}(t)$, needed to compute the correlation matrix and the r.h.s. of Eq. 3, obeys for a linear rate function r(x)=x,


(20) $$\frac{d{\bar{x}}^{\left(l\right)}}{dt}+{\bar{x}}^{\left(l\right)}-gM{\bar{x}}^{\left(l\right)}=f(t)b=b\sum_{n=1}^{M}f_{n}e^{i\omega_{n}t},$$

where we have introduced the frequency decomposition of the function *f* to be learnt, with $f_{m}=f_{n}^{*}$ when $\omega_{m}=-\omega_{n}$, for f(t) to be real-valued. The forced solution is readily obtained as,


(21) $${\bar{x}}^{\left(l\right)}(t)=\sum_{n}A_{n}e^{i\omega_{n}t}u_{n}^{\left(l\right)},\;\mathrm{with},$$

(22) $$u_{n}^{\left(l\right)}=[(1+i\omega_{n})I_{N}-gM{]}^{-1}b.$$

It provides an explicit expression of Eq. 3 for w,


(23) $$\rho w+\sum_{n=1}^{M}|f_{n}{|}^{2}\;u_{n}^{\left(l\right)}(u_{n}^{(l)*T}w)=\sum_{n=1}^{M}|f_{n}{|}^{2}\;u_{n}^{\left(l)\right)},$$

where * denotes complex conjugation. The vectors $u_{n}^{\left(l\right)}$ only span a subspace *U* of dimension *M* of the whole activity space of dimension N>M. Therefore, the correlation matrix is of rank *M* and w is underdetermined without regularization (ρ=0). With a nonzero regularization (ρ≠0), Eq. 23 shows that w is a linear combination of the vector $u_{n}^{\left(l\right)}$. We can write it as


(24) $$w=\sum_{n=1}^{M}a_{n}u_{n}^{\left(l\right)}.$$

The $a_{n}$ are the solution of the M×M system obtained by identifying the coefficients of each $u_{n}^{\left(l\right)}$ on the two sides of Eq. 23,


(25) $$a_{n}\;\rho /|f_{n}{|}^{2}+\sum_{m=1}^{M}u_{n}^{(l)*T}u_{m}^{\left(l\right)}\;a_{m}=1.$$

The regularization *ρ* eliminates the indeterminacy of the vector w by restricting it to the subspace spanned by the vectors $u_{n}^{\left(l\right)}$. Once Eq. 24 is imposed, the very small *ρ* in Eq. 25 has a negligible effect for a small number of modes *M* and the first term in Eq. 25 can be dropped. Equation 25 then simply reduces to the conditions $w^{T}u_{n}^{\left(l\right)}=1,\;n=1,\ldots ,M$ which implies that the $u_{n}^{\left(l\right)}$s are eigenvectors of L (Eq. 4). As a consequence, Eq. 21 for ${\bar{x}}^{\left(l\right)}(t)$ does not only provide a solution of the forced dynamics but also one of the autonomous linear dynamics. The projection of this linear solution by the readout w approximates the function f(t) when the amplitudes $A_{n}$ in Eq. 21 are the amplitudes of the Fourier modes of f(t), $A_{n}=f_{n}$.

### Explicit computation of normal forms

We detail here the computation of normal form equations and coefficients for the example of the single frequency sine function (Eq. 7). As stated in the main text, after learning, L has two c.c. eigenvalues $\lambda_{1}$ and $\lambda_{1}^{*}$ close to iω and −iω, with right eigenvectors $u_{1}$ and $u_{1}^{*}$ and left eigenvectors $v_{1}^{T}$ and $v_{1}^{*T}$,


(26) $$Lu_{1}=\lambda_{1}u_{1},v_{1}^{T}L=\lambda_{1}v_{1}^{T},$$

with the corresponding equations for $u_{1}^{*},v_{*}$, and $\lambda_{1}^{*}$. The eigenvectors obey the orthogonality relations $v_{1}^{*T}u_{1}=v_{1}^{T}u_{1}^{*}=0$, and we choose the normalization conditions $v_{1}^{T}u_{1}=v_{1}^{*T}u_{1}^{*}=1$. We search in perturbation for a small amplitude solution of Eq. 1 of the form $x=[A_{1}(t)e^{i\omega t}u_{1}+c.c]+x_{p}$. The amplitude $A_{1}$ evolves on a slow time scale controlled by the small real part of $\lambda_{1}$, $\lambda_{1}^{R}∼\epsilon \ll 1$, while $|A_{1}|∼\epsilon^{3/2}$ and $x_{p}∼\epsilon^{3/2}$. Substitution into Eq. 1 gives for $x_{p}$,


(27) $$\begin{array}{c} \frac{dx_{p}}{dt}-Lx_{p}+\left[\frac{dA_{1}}{dt}e^{i\omega t}u_{1}+c.c\right]=(\lambda_{1}-i\omega )A_{1}u_{1}\\ \;+r_{3}[gM+bw^{T}][3|A_{1}{|}^{2}A_{1}e^{i\omega t}u_{1}^{2}u_{1}^{*}+\cdots +c.c.], \end{array}$$

where the second term on the r.h.s. comes from the expansion of the rate function and the vector power is meant component-wise. We have only explicitly written the lowest-order “resonant” term proportional to exp(iωt). It gives rise to a secular term in $x_{p}$ unless $x_{p}$ has no component on $u_{1}$ when written in the basis of the (right) eigenvectors of L. In other terms, it should be orthogonal to $v_{1}$. Multiplying both sides of Eq. 27 on the left by $v_{1}^{T}$ gives Eq. 5 with the expression 6 for the constant $g_{A}$.

A similar computation provides the expressions of the coefficients $g_{ij}$ for the normal form in the two-frequency case (Eq. 11),


(28) $$g_{A,ij}=3r_{3}(2-\delta_{ij})(\lambda_{j}+1)v_{j}^{T}\left[|u_{i}{|}^{2}u_{j}\right],\;g_{i,j}=\frac{g_{A,ij}}{|w^{T}u_{i}{|}^{2}},$$

where the us and vs are the right and left eigenvectors of L associated to the eigenvalues $\lambda_{1}$ and $\lambda_{2}$, as indicated by their subscript. They are normalized such that $v_{1}^{T}u_{1}=\delta_{ij}$, where here and in Eq. 28, we have used the Kronecker symbol, $\delta_{ij}=1$ if i=j, and 0 otherwise.

### Universal coefficients for N→∞ and $f_{1}\to 0$

In the limit of large recurrent networks with random interaction matrices M, several quantities become independent of the particular choice of M. This is in particular the case, for the coefficients $a_{i}$ which determine the readout vector w, and, most importantly, for the coefficients in the normal form equations.

We consider first the determination of w (Eq. 24)) that positions *M* eigenvalues of the matrix L (Eq. 4) at values $i\omega_{m}$ on the imaginary axis. The vector w is a linear combination of the corresponding eigenvectors $u_{m}^{\left(l\right)}$ (Eq. 22) with coefficients $a_{m}$, solutions of a system of linear equations with coefficients $u_{n}^{(l)T}u_{m}$ (Eq. 25). We expand rational functions of M in power series in order to compute their limiting values in the limit N→∞,


(29) $$u_{m}^{\left(l\right)}=\frac{1}{\sigma_{m}}\sum_{k=0}^{+\infty }{\left(\frac{g}{\sigma_{m}}\right)}^{k}M^{k}b,\;\mathrm{with},\;\sigma_{m}=1+i\omega_{m}.$$

Averaging with respect to the elements of M, with zero mean and a variance equal to 1/N, gives the mean and correlation of the eigenvectors,


(30) $$\langle u_{m}^{\left(l\right)}\rangle =\frac{1}{\sigma_{m}}b,\;\langle u_{n}^{(l)T}u_{m}^{\left(l\right)}\rangle =\frac{b^{T}b}{\sigma_{n}\sigma_{m}-g^{2}},$$

where we have used that at dominant order $\langle M_{i,l_{1}}^{k_{1}}M_{j,l_{2}}^{k_{2}}\rangle =\delta_{i,j}\delta_{k_{1},k_{2}}\delta_{l_{1},l_{2}}/N$. The coefficients $a_{m}$ (Eq. 24), when N→∞ and ρ→0 are solution of the linear system (Eq. 25),


(31) $$\sum_{m=1}^{M}\frac{a_{m}}{\sigma_{n}\sigma_{m}-g^{2}}=\frac{1}{b^{T}b}.$$

Inversion of this linear system ^d^ provides the limiting values of the coefficients $a_{m}$ when N→∞,


(32) $$a_{m}=g^{2(1-M)}\frac{\prod_{n,n\neq m}\sigma_{n}\;\prod_{n=1}^{M}(\sigma_{m}\sigma_{n}-g^{2})}{b^{T}b\;\prod_{n,n\neq m}(\sigma_{m}-\sigma_{n})}.$$

Equation 32 fully determines the read out vector w and, for instance, determines its norm as a function of the imposed eigenvalues $i\omega_{m}$. The conditions $w^{T}u_{m}^{\left(l\right)}=1$ imply that the square norm of w is given by the sum of the $a_{m}$,


(33) $$w^{T}w=\sum_{m=1}^{M}a_{m}w^{T}u_{m}^{\left(l\right)}=\sum_{m=1}^{M}a_{m}.$$

The last term is equal to the sum of all residus, except the one at the origin (z=0), of the rational function $F(z)=g^{2(1-M)}$  $\prod_{\;j=1}^{M}(z\sigma_{j}-g^{2})/[zb^{T}b\prod_{\;j=1}^{M}(z/\sigma_{j}\;-1)]$. Since $F(z)∼g^{2(1-M)}$  $(\prod_{\;j=1}^{M}\sigma_{j}^{2})/z$ when z→∞, its integral around a large circle is both equal to $g^{2(1-M)}(\prod_{\;j=1}^{M}\sigma_{j}^{2})$ and to the sum of all its residus (including the one at zero). Thus, one obtains,


(34) $$w^{T}w=\frac{g^{2(1-M)}}{b^{T}b}\prod_{\;j=1}^{M}\sigma_{j}^{2}-\frac{g^{2(1-M)}}{b^{T}b}=\frac{g^{2}}{b^{T}b}\left[-1+\prod_{\;j=1}^{M}\frac{\sigma_{j}^{2}}{g^{2}}\right].$$

Equation 34 shows that the norm of the rank 1 feedback diverges with the number *M* of eigenvalues, when one takes $\omega_{m}=m\omega$, which limits the number of eigenvalues that can be prescribed. We also note that Eq. 34 is similar to a numerically based conjecture in Ref. (22) proposed in a different modeling framework.

With the vector w determined (Eqs. 24 and 32), the coefficients of normal form equations (eg Eq. 28) in the small feedback limit can be computed from the knowledge of the right and left eigenvectors of L (Eq. 4) for a linear rate function. The right eigenvectors for imposed eigenvalues $i\omega_{m}$ are given by Eq. 22 and the corresponding left (nonnormalized) eigenvectors are given by similar expressions,


(35) $$v_{m}^{(l)T}=w^{T}[(i\omega_{m}+1)\;I_{N}-gM){]}^{-1}.$$

We illustrate this computation for the example of the one-frequency case, ie for a sine function of very small amplitude ($f_{1}\to 0$) and frequency $\omega_{1}=\omega$. The expression of the coefficient $g_{1}$ (Eq. 9) is obtained from Eq. 7 with $u_{1}=u_{1}^{\left(l\right)}$ (Eq. 22) and $v_{1}=v_{1}^{\left(l\right)}/(v_{1}^{(l)T}u_{1}^{\left(l\right)})$ (Eq. 35), where the denominator enforces the chosen normalization of $v_{1}$. The vector w is simply given as (Eq. 24), $w=a_{1}u_{1}^{\left(l\right)}+c.c$, with $a_{1}$ equals to (Eq. 32),


(36) $$a_{1}=\frac{-i}{2g^{2}\omega \;b^{T}b}[1-i\omega ][1+\omega^{2}-g^{2}][(1+i\omega {)}^{2}-g^{2}].$$

The scalar products appearing in the numerator and denominator of $g_{1}$ (Eqs. 6 and 7), can be computed by expanding rational function of M in powers as above (Eqs. 29 and 30; note that $g_{1}=g_{A}$ for small forcing amplitudes since then $w^{T}u_{1}=1$). One obtains


(37) $$\begin{array}{rl} v_{1}^{(l)T}[|u_{1}^{\left(l\right)}{|}^{2}u_{1}^{\left(l\right)}] & =\frac{4i\omega (1+\omega^{2})}{[(1+i\omega {)}^{2}-g^{2}][1+\omega^{2}-g^{2}{]}^{2}}\frac{\left(b^{T}b\right)}{N}\\ v_{1}^{(l)T}u_{1}^{\left(l\right)} & =\frac{2i\omega (1+\omega^{2})}{\left[(1+i\omega {)}^{2}-g^{2}][1+\omega^{2}-g^{2}\right]}. \end{array}$$

Substitution of these expressions in Eq. 6 finally provides the expression for $g_{1}$ (Eq. 9) in the main text. We leave it for further studies but note that the variance of $g_{1}$ could be obtained by similar computations. This would provide a precise analytical estimate for the concentration phenomenon reported in Fig. 2f.

## Data Availability

There is no data underlying this work.

## Notes

a We note that Eq. 1 is usually called a “rate” or “neural mass” model in the neuroscience literature (12) and is here formulated in the usual version (6, 7) where the variables $x_{i}$ are meant to represent the membrane potential of unit *i*. An alternative version where the variables are the neuron discharge rates is often used in the ESN literature (1). Both are mathematically equivalent as shown in Materials and methods section.

b A previous study of this case, purely at the linear level, is given in Ref. (23).

c A subtlety here is that the relative phase $2\phi_{1}-\phi_{2}$ of the two modes is arbitrary for the lowest order Eq. 11. It is only fixed by higher order terms, respecting the required symmetries such as $Z_{2}^{2}Z_{1}^{*3}$ in the $Z_{1}$ equation or $Z_{1}^{4}Z_{2}^{*}$ in the $Z_{2}$ equation. Correlatively, when the network dynamics is perturbed after learning, it returns extremely slowly to its correct values when $f_{1}$ and $f_{2}$ are small.

d We make use of the following two determinant formulas. For the matrix A with $A_{ij}=1/(\alpha_{i}+\beta_{j})$, $\det A=\prod_{i<j}(\alpha_{i}-\alpha_{j})\prod_{i<j}(\beta_{i}-\beta_{j})/\prod_{i,j}(\alpha_{i}+\beta_{j})$. Similarly, the determinant of the matrix $A_{1}$, deduced from A by replacing its first column by a column of 1, reads $\det A_{1}=\prod_{i<j}(\alpha_{i}-\alpha_{j})\prod_{2\leq i<j}$  $\left(\beta_{i}-\beta_{j})/\prod_{1\leq i,2\leq j}(\alpha_{i}+\beta_{j}\right)$.

## Preprints

This manuscript was posted as a preprint: https://doi.org/10.1101/2025.06.23.661158.
